# Association of Personality Traits with Life and Work of Medical Students: An Integrative Review

**DOI:** 10.3390/ijerph191912376

**Published:** 2022-09-28

**Authors:** Meichen Liu, Jinquan Cai, Hao Chen, Lei Shi

**Affiliations:** 1 Modern Educational Technology Center, Harbin Medical University, Harbin 150086, China; 2 Department of Neurosurgery, The Second Affiliated Hospital of Harbin Medical University, Harbin 150086, China; 3 School of Health Management, Southern Medical University, Guangzhou 510515, China

**Keywords:** personality traits, medical students, life, work

## Abstract

Background: Personality traits are the basic components of an individual’s personality. Although there are many published articles about the impact of personality traits on medical students, there is a lack of integrative reviews of existing articles. To close this gap, this review aims to summarize the impact of personality traits on medical students from two perspectives: life and work. Methods: The search was performed using the following databases: PubMed, Web of Science, Google Scholar, and EMBASE. All publications that assessed the impact of personality traits on life and work until February 2022 were selected. Results: Ninety-seven studies were included. The results suggest that personality traits could affect life performance, health outcomes, life satisfaction, the formation of doctor–patient relationships, mastery of knowledge, academic performance, and career planning. Different personality traits can have positive or negative impacts on these aspects. Conclusions: The results of this review suggest that personality traits can affect medical students’ lives and work. Therefore, based on the evaluation of the personality traits of medical students, it is necessary to design targeted courses and training for students to improve their personality traits, to bring about better results in their lives and work.

## 1. Introduction

Personality traits are important psychological characteristics and effective predictors of personal behavior and results [1]. The Big Five is a method of classifying personality traits which developed in the field of psychological trait theory in the last century. At present, almost all personality measurements are classified based on the five-factor personality model. The dimensions of these five characteristics are neuroticism, extroversion, openness, agreeableness, and conscientiousness [2]. The labels for the five factors can be remembered using the acronym “OCEAN” or “CANOE”. At present, there are several different ways to measure the Big Five personality traits. As examples, take the International Personality Item Pool (IPIP), NEO-PI-R, The Ten-Item Personality Inventory (TIPI), and the Five Item Personality Inventory (FIPI). The current research shows that personality traits can widely affect the education, work, political identities, and even religious beliefs of different people (including medical students). At present, the research in this field focuses on exploring the specific mechanisms of the influence of personality traits on individual behavior and how to improve the accuracy of personality traits’ measurement.

Personality traits mainly affect medical students in two aspects: life and work. Life performance, health outcomes, and satisfaction constitute parts of life that personality traits affect. Personality traits can affect health outcomes via health risk perception or indirectly affect health outcomes through life performance (e.g., physical activity, sleep, diet, smoking, and drinking). Personality traits have been proven to be significant predictors of poor sleep [3], future drinking, and future smoking [4]. Personality traits are also related to different levels of physical activity, and the relationship between them does not change with age or sex [5]. The differences in eating habits were also considered to be related to personality traits [6,7]. Health risk perception, defined as people’s subjective judgment of the characteristics and severity of their health risk, is also affected by personality traits [8,9]. In addition, life satisfaction is regarded as an indicator of people’s happiness and is also influenced by personality traits [10].

Doctor–patient relationships, mastery of medical knowledge and clinical skills, career planning, and academic performance are all important branches influenced by medical students’ personality traits. The importance of the doctor–patient relationship should be fully acknowledged and properly valued during medical education. Empathy and appropriate medical communication skills are key to maintaining a good doctor–patient relationship [11]. According to recent research, both of these are related to personality traits. Therefore, shaping certain personality traits is necessary to improve medical students’ empathy and communication skills and obtain a good doctor–patient relationship. In the changing learning context provided by a five-year medical degree, the same personality trait might have different influences on medical students’ mastery of medical knowledge and clinical skills [12]. Teaching methods should be altered according to different learning stages to respond to this change. Personality traits have also been proven essential for medical students to choose the most suitable specialties [13]. Therefore, medical educators should focus on guiding undergraduates to make the best choices according to their personality traits. Additionally, certain personality traits have been considered the best predictors of academic education and clinical training success.

Many studies have shown that personality traits can affect the life and work of various groups, including medical students; however, most of the current articles about the impacts of personality traits only focus on one aspect of life or work (such as life performance, health outcomes, life satisfaction, doctor–patient relationship, medical knowledge, clinical skills, academic performance or career planning). There is a lack of an integrative review of this phenomenon that personality traits have an impact on many aspects of life and work. In addition, some studies on these influences in other articles only discussed the roles of certain personality traits on individuals in depth, but neglected the research on other aspects of the five personality traits. Therefore, in view of the above research, we collected relevant articles as widely as possible, and reviewed the comprehensive impacts of various aspects of personality traits on medical students. Finally, we believed that personality traits not only had a wide impact on the life and work of medical students, but also that some personality traits had opposite effects on medical students in different periods. Therefore, it is a meaningful research direction for medical educators to design targeted courses and training for students according to their different personality characteristics to improve medical students’ beneficial personality traits and their performance in life and work.

## 2. Literature Search

### 2.1. Search Strategy

**Figure 1:**
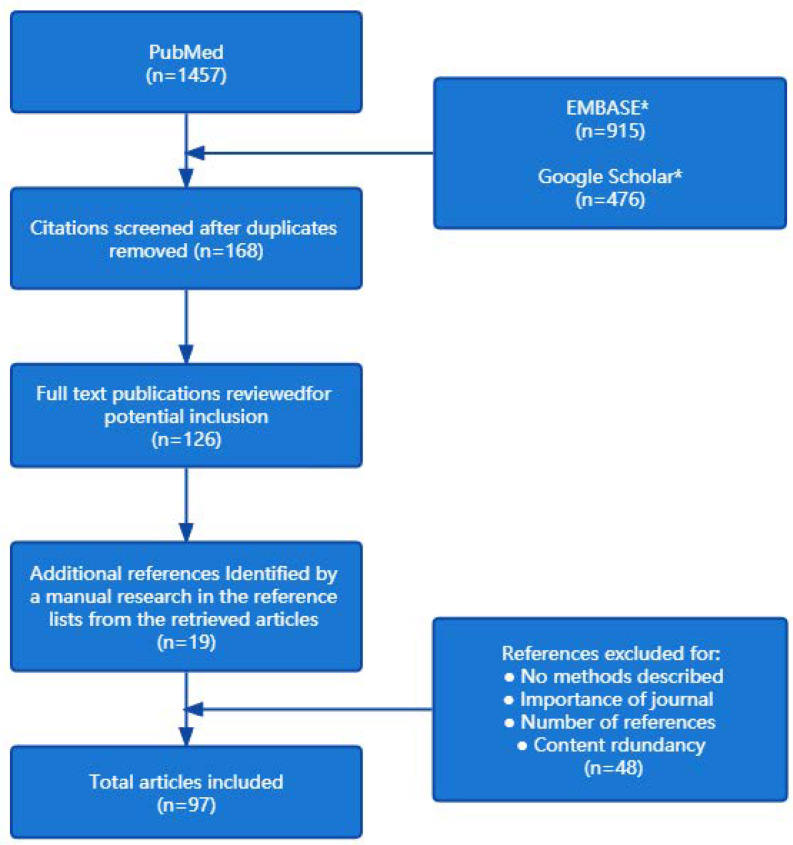
Flowchart of the literature selection. * Google Scholar and EMBASE were auxiliary databases used if the full text of a particular article was not found in PubMed.

The electronic literature retrieval of this review was mainly carried out through PubMed, and if the full text of an article could not be found in PubMed, Web of Science, EMBASE, and Google Scholar were secondary databases. The search string included the following keywords: “personality traits” OR “Big Five personality dimensions” AND “life performance” OR “health outcome” OR “life satisfaction” OR “doctor–patient relationship” OR “medical knowledge” OR “clinical skills” OR “career planning” OR “academic performance” AND “medical students”. After removing duplicates, abstracts were independently analyzed by two reviewers. Conflicts of opinion were resolved by discussion with a third reviewer. Finally, the full texts were read and checked by two reviewers, who chose the studies to be included in the review. All relevant articles published from 1997 to 2022 were retrieved, and the last search was conducted on February 17, 2022. The keywords were commanded to appear in “title” and “title and abstract”. The search process is illustrated in the flowchart (Figure 1).

### 2.2. Inclusion Criteria

Articles included in this review, regardless of publication date and magazine, had to meet the following inclusion criteria: subjects in the experiment should accept at least one kind of personality trait test; articles must have been published within the past 25 years (1997–2022); should emphasize the relationship between personality traits and personal life and work; and should be peer-reviewed journal articles written in English.

### 2.3. Exclusion Criteria

The exclusion criteria were as follows: editorials, letters to the editor, viewpoints, case presentations, grey literature, brief communications, pilot studies, conference papers, articles not written in English, and those for which we could not obtain the full text. Articles with redundant content and lacking research methods and references were also excluded. 

### 2.4. Data Collection

Data extraction included the author, publication date, country, title of the study, aim, study type, sample, median or mean age (years)/sex, methods/scale, and observations. Two reviewers independently performed the extractions. The methodological characteristics of the studies included in this review are described in Table S1 (see the Supplementary Material for details). 

Additionally, by searching the references of the articles included in this review in the first round of the search, we found other articles that met the standard. Through the same evaluation steps, we included qualified parts of the newly identified articles in this review. 

## 3. Influence of Personality Traits on Medical Students

### 3.1. Life Performance 

This differences in personal psychology and behavior caused by personality traits are considered to be related to individual differences in life performance. Personality traits are associated with various manifestations, such as physical activity (PA), sleep, diet, smoking, and drinking. Sutin et al. examined the relationships between personality traits and physical inactivity, the frequency of physical activity, and sedentary behavior. They found that these traits might affect the level of physical activity required to determine whether a person has a sedentary lifestyle. Higher neuroticism and lower conscientiousness indicate more time spent in actual sedentary behaviors. Lower neuroticism and higher conscientiousness were associated with more physical activity, and extraversion and openness were also associated with more physical activity and less inactivity [5]. Extraversion was consistently associated with a greater frequency of physical activity because extraverted individuals could enjoy an increase in positive emotions after engaging in physical activity [14]. There is little evidence that the relationship between physical activity and personality traits changes with age or gender. Therefore, personality traits may be a considerable obstacle or promoter of physical activity [5]. 

Additionally, five personality traits could also be associated with sleep variables, including sleep duration and sleep quality. A recent study showed that higher extraversion, agreeableness, and conscientiousness are associated with better sleep quality, whereas people with higher neuroticism usually slept less. There is insufficient evidence regarding the relationship between openness and sleep [15]. In Duggan et al.’s study, valid self-report measures of personality, chronotype, sleep hygiene, sleep quality, and sleepiness were analyzed. They found that low conscientiousness and high neuroticism were the best predictors of poor sleep (poor sleep hygiene, low sleep quality, and increased sleepiness) [3]. In general, it could be considered that personality traits were highly correlated with sleep. The higher the neuroticism, the worse the sleep. In contrast, the higher the conscientiousness, the better the sleep. 

In addition to sleep, personality traits can influence eating habits. More relevant studies have shown that individuals with high openness tend to eat more fruits and vegetables and consume less sugar [7]. After controlling for age and gender, Weston et al. found that people with higher levels of agreeableness, conscientiousness, and openness and lower levels of neuroticism tended to consume more fruits and vegetables than junk foods [16]. In contrast, high levels of neuroticism often suggest an excessive intake of traditional convenience food (eating more tinned vegetables, meat pies, sausage rolls, puddings, etc.) [6]. Keller et al. also confirmed that high openness was associated with higher fruit, vegetable, and salad consumption and lower meat and soft drink consumption [7]. In summary, personality traits have been proven to be related to dietary habits. It is helpful that we intervene and optimize people’s eating habits by identifying people’s personality traits and subsequently predicting the corresponding eating habits. 

Additionally, some studies have indicated that smoking is associated with personality traits. One project examined the relationships between personality traits and lifetime smoking, daily cigarette consumption, and smoking persistence among adults in the United States over ten years. Using logistic regression, this research revealed that higher levels of openness and neuroticism were associated with an increased risk of daily smoking and smoking persistence; conversely, conscientiousness had the opposite effect on smoking [17]. Kulkarni et al. investigated the associations between the level of nicotine dependence, personality traits, and smoking behavior among Indian smokers working in the corporate sector. They found that neuroticism was significantly associated with the level of nicotine dependence and more smoking [18]. Moreover, according to a study of 1897 youth aged 11 to 15 years in the United States, researchers suggested that low conscientiousness could predict future high school students’ drinking and smoking, and they also noted that the effect was independent of gender and race [4]. 

Personality traits have also been associated with alcohol consumption. Malouff et al. studied the relationships between the five personality traits and alcohol involvement through a meta-analysis of 20 studies with 119 effect sizes and 7886 participants. They concluded that alcohol involvement was associated with low conscientiousness, low agreeableness, and high neuroticism [19]. Another study that examined the impact of women’s personality traits on their drinking habits before and during pregnancy reached a similar conclusion. The researchers also believed that high conscientiousness and agreeableness were protective factors against alcohol consumption during pregnancy, whereas women with high extraversion were more likely to consume alcohol [20]. 

The aforementioned studies on personality traits and life performance were not conducted on medical students. Even so, these conclusions still have certain implications for the concrete embodiment of the relationship between personality traits and the life performance of medical students. According to the detectable personality traits, how to intervene and guide the life performance of medical students, to urge them to make full preparations for their future study and life, still needs further research, and research results might be of considerable value.

### 3.2. Health Outcomes

Personality traits may be directly and/or indirectly (via health-related life performance) associated with health outcomes. It is suggested that the direct influence of personality traits on health outcomes is related to health risk perception, which means that people might react to specific health risks in line with their personality traits. A cross-sectional study investigated the relationship between personality traits and diabetes mellitus type 2 (T2DM) risk perception among university students in Denmark. This study revealed that higher levels of conscientiousness and emotional stability were directly negatively associated with T2DM risk perception after adjusting for health-related life performance and body mass index (BMI) [21]. In addition, Vollrath et al. studied the relationships among personality traits, health-related life performance, and perceptions of susceptibility to health risks among 683 university students. They suggested that agreeableness and conscientiousness negatively influenced risk perceptions of susceptibility to lung cancer, alcohol dependency, and venereal disease, and neuroticism was the only personality trait that showed positive direct effects on risk perceptions of susceptibility [22]. In summary, agreeableness and conscientiousness negatively affected health risk perceptions, whereas neuroticism had positive effects. Although there is a relationship between personality traits and risk perception, actual health outcomes are also related to life performance. 

Personality traits can also indirectly affect health outcomes by affecting life performance. Sleep duration and quality, influenced by personality traits, are common research topics for the healthy. Recently, a systematic review was performed based on the duration of sleep and other inclusion criteria of BMI, the prevalence of obesity, age, and sex, and it was observed that short sleep duration (due to high neuroticism) would cause a consistently increased risk of obesity [23]. Higher neuroticism usually triggers chronic sleep loss, which might represent a novel risk factor for weight gain, insulin resistance, and type 2 diabetes [24]. Physical inactivity, such as sedentary activity induced by high neuroticism, was also associated with elevated risks for obesity, cardiovascular disease, type 2 diabetes, breast and colon cancers, and mortality [25,26]. Regarding the effects of personality traits on health outcomes through eating habits, Bailey et al. suggested that the prevalence of diabetes with low fruit and vegetable consumption and low physical activity was >50% [27]. Higher openness was related to eating more fruits and vegetables, indicating a lower risk of diabetes [28]. 

In addition, personality traits may also affect health outcomes by influencing habits (e.g., smoking and drinking). Mackenbach et al.’s study gave new insights into the effects of smoking on health: the risk of premature death was three times as high in smokers as in non-smokers, and the mechanisms of cancer, ischemic heart disease, and nicotine dependence under the influence of smoking were greatly clarified [29]. Thus, smoking caused by high neuroticism affects the health of both smokers and those exposed to tobacco smoke. Furthermore, the International Agency for Research on Cancer has classified alcohol as a group 1 carcinogen. Epidemiologists have considered the relationships between alcohol consumption and oral, esophageal, gastric, liver, and colon cancers [30,31]. Therefore, excessive drinking caused by low conscientiousness, low agreeableness, and high neuroticism is highly correlated with the occurrence of cancer.

BMI is widely used to analyze the health status of individuals quantitatively. Sutin et al.’s study analyzed self-reported measures of personality traits, physical activity, diet and food intake behavior, height, and weight. They found that a high level of neuroticism was associated with a higher BMI and the risk of obesity, whereas conscientiousness, extraversion, and openness had the opposite effects. Physical activity is a key factor in the links between personality traits and BMI [32]. Moreover, a large longitudinal study (*n* = 1988) spanning more than 50 years examined how personality traits were associated with fluctuations in BMI. This suggests that high neuroticism and low conscientiousness are the personality traits most relevant to BMI and obesity [33]. Neither high nor low BMI has a beneficial effect on physical health. Excessive BMI is associated with increased risks of lifestyle-related diseases, including heart disorder [34], cerebrovascular disease [35,36], and diabetes [37]. Low BMI is related to increased risks of undernutrition [38], amenorrhea, osteoporosis, and osteopenia [39]. In addition, gender can affect the relationship between extraversion and BMI. Extraversion was negatively associated with BMI among women but was unrelated to BMI among men [32]. However, previous studies found an opposite conclusion: men who scored higher on extraversion tended to weigh more, whereas extraversion was unrelated to BMI among women [40]. This difference might have been due to the different scales used to measure extraversion in the different studies. In other studies, openness and agreeableness either have a negative relationship [41] or no relationship [42] with BMI. Evidently, neuroticism and conscientiousness could better predict an individual’s BMI status than other traits, suggesting that personality traits affect health outcomes through BMI. 

At present, neurodegenerative diseases (such as Alzheimer’s disease and Parkinson’s disease) have new treatments, such as clinical cell therapies [43] and subthalamic nucleus deep brain stimulation [44], but how to evaluate the risk still has practical significance for disease prevention and treatment. The test of personality traits provides a new idea for it. D’Iorio et al. conducted a systematic literature search through sycInfo (PROQUEST), PubMed, and Scopus. After meta-analysis, they believed that high levels of neuroticism, low openness, and low extroversion were conducive to the long-term progress of Alzheimer’s disease [45]. In addition, other researchers found similar conclusions on the relationships between personality traits and the risk of Parkinson’s disease: neuroticism was associated with an increased risk of Parkinson disease [46].

The subjects involved in the aforementioned studies might not have had a medical education background. However, medical students can master more personality traits and health knowledge than ordinary people without a medical background, which may cause them to apply their professional knowledge to maintain their health. Compared with the above conclusions drawn from other groups, the specific relationship between personality traits and health outcomes among medical students may differ. This possible difference merits further investigation. 

### 3.3. Life Satisfaction

Satisfaction with life is “a global assessment of a person’s quality of life according to their chosen criteria” [10]. It is a construct from the domain of positive psychology, including cognitive, affective, and behavioral dimensions. A series of studies indicate that many factors could affect the self-assessment of life satisfaction in young people [47]. Several variables, including socio-demographic features (age, gender, educational level, and marital status), health, and income, had a negligible role in explaining the variance in satisfaction with life [48]. Personality traits might affect medical students’ psychological adaptation to the real world, and their life satisfaction [48]. Another study has revealed that personality traits are significantly associated with variance in satisfaction with life in young people [49]. Personality traits such as neuroticism and extroversion have been found to be predictors of life satisfaction [50]. Different personality traits and satisfaction with life scales can be substantively different when the relationship between personality traits and satisfaction with life is analyzed. Neuroticism and extraversion are nearly identical to the two elements of subjective well-being (SWB, including life satisfaction), negative and positive effects, respectively. Neurotic individuals might become anxious, easily upset, or depressed, whereas extraverts tend to be optimistic, outgoing, energetic, expressive, active, assertive, and exciting [51]. Navarro-Prados et al. collected the information on personality traits and life satisfaction of 342 participants through self-reported questionnaires. After statistical analysis, they believed that the results proved relationships between personality characteristics and life satisfaction: extroversion, agreeableness, and conscientiousness were positively correlated with life satisfaction, whereas neuroticism was negatively correlated [52]. Another study conducted among adolescents reached the same conclusion [53]. 

It has been reported that medical students’ level of satisfaction with life decreased from their first to the third year of medical school and remained at a lower level until graduation; the comparison results showed that medical students had a level of satisfaction with life similar to that of other students when beginning their medical studies, but reported a lower level than the control group in their final learning year [54]. Perceived medical school stress has been associated with mental distress and forthcoming mental health problems, and its effect on life satisfaction is therefore assumed. We should study the personality traits of students who sustained high levels of life satisfaction during medical school and compare them with the personality traits of their peers to find characteristics that may be used to make positive changes and hence help improve the satisfaction with life of medical students [55]. The students with high life satisfaction scored lower on the personality trait vulnerability (the neuroticism dimension), had less academic worries, perceived medical school as interfering less with their social and personal lives, were more likely to cope with stress by using a problem-focused approach and seeking social support, and were less likely to turn to wishful thinking [54]. A 10-year longitudinal study revealed that a low level of neuroticism was a significantly adjusted predictor of life satisfaction in Norwegian doctors in their ninth postgraduate year, but the increase in life satisfaction from T1 to T2 was predicted by lower levels of conscientiousness [56]. In summary, medical schools could encourage students to balance learning work and their social and personal lives, focus on their health status, and help modify their personality traits, stress, and coping.

### 3.4. Doctor–Patient Relationship

For medical students, personality traits can affect health and life and doctor–patient relationships. Pursuing good doctor–patient relationships should be regarded as one of the lifelong goals of each medical student after participating in clinical work. A good and effective doctor–patient relationship depends on empathy, clinical communication skills, and emotional intelligence, all of which are influenced by personality traits [11].

Medical communication, based on empathy, is defined as the ability to understand patients’ experiences and concerns [57], and respond to appropriate emotions [58,59,60]. According to this concept, doctors with empathy can gain patients’ trust, improve their compliance, and form good relationships with patients, finally achieving the best results in clinical practice [61,62,63]. Conversely, communication without empathy might deteriorate the doctor–patient relationship [64]. Recently, Wang et al. selected 2665 doctors and 2983 patients and examined the effect of doctor empathy on doctor–patient relationships and the intermediary role played by doctor communication between doctors’ empathy and doctor–patient relationships. Finally, they concluded that enhancing doctor empathy helped improve the communication efficiency of doctors, which ultimately became the key to constructing a harmonious doctor–patient relationship [65]. Progress has been made in understanding how personality traits affect empathy. Some studies have investigated the relationship between personality traits and empathy. After conducting a statistical analysis, a study on the relationship between personality traits and empathy scores confirmed positive association between agreeableness, openness, and empathy, but it did not support the researchers’ hypothesis of negative associations between neuroticism and empathy [66]. 

Another study on the NEO Five Factor Inventory (NEO-FFI) and the Jefferson Scale of Doctor Empathy (JSPE-spv) among 472 medical students also verified the above conclusion: the positive associations between agreeableness and openness and empathy of medical students. In addition, this study stated that there was no significant link between empathy and conscientiousness [67]. Additionally, other researchers suggested that good communication skills could improve patients’ satisfaction with care and physical health and ameliorate the doctor–patient relationship [68]. Therefore, effective doctor–patient communication skills are essential skills for medical students who pursue good doctor–patient relationships. One study collected the results of the Communication Skills Attitude Scale (CSAS-BR) and the Big Five Mini-Markers (BFMM) for personality from Brazilian college students. After further analysis, the researchers believed that agreeableness, openness, and extroversion were the most positive factors affecting students’ communication abilities [69]. Based on these statements, agreeableness, openness, and extroversion could form good doctor–patient relationships by improving the communication ability of medical students. In addition to empathy and clinical communication skills, emotional intelligence influenced by personality traits is another factor that can affect the doctor–patient relationship. An increased level of emotional intelligence was shown to positively influence doctor–patient relationships [70]. Recently, a study focused on assessing the relationship between emotional intelligence and personality traits among American medical students. This study revealed that emotional intelligence was positively correlated with extraversion, conscientiousness, agreeability, and openness and negatively correlated with neuroticism [71]. Another cross-sectional questionnaire-based study conducted at an Irish medical school verified the same conclusion: extroversion, conscientiousness, agreeability, and openness are related to high emotional intelligence [72]. According to the above research conclusions, medical students with high levels of extroversion, conscientiousness, agreeability, and openness were more likely to maintain a good doctor–patient relationship because of their high emotional intelligence.

According to the above research results, extroversion, conscientiousness, agreeability, and openness may improve medical students’ empathy, emotional intelligence, and communication skills, and finally, help medical students maintain good doctor–patient relationships with patients. Medical training should focus on improving these personality traits (extroversion, conscientiousness, agreeability, and openness). Nevertheless, this cannot be achieved without a proper assessment of personality traits. Therefore, before designing these courses, it is necessary to understand students’ personality traits to provide suitable training. 

### 3.5. Medical Knowledge and Clinical Skills

In the changing learning context provided by the five-year medical degree, the same personality traits may have different effects on medical students’ mastery of their skills and knowledge. In the first three pre-clinical years, medical students mainly acquired medical knowledge through standard teaching courses, with a test by examination. In the final two clinical years, medical students learn clinical skills (doctor–patient communication, physical examination, disease diagnosis, medication, or operation) [73,74]. They then gradually come into contact with a large number of patients and need to use their knowledge and skills to diagnose and treat patients. Finally, the student’s clinical skills are assessed using an objective structured clinical examination (OSCE). 

Ferguson et al. examined the associations between the Big Five personality traits and learning outcomes (including medical knowledge and clinical skills) across five years of a medical degree among 220 UK medical students [12]. They found that the relationships between personality traits and learning outcomes might change in different learning contexts, from pre-clinical to clinical years. Researchers found a U-shaped relationship between extraversion and mastery of medical knowledge in the first three pre-clinical years. This relationship reflected that low-level extraversion was related to better mastery of medical knowledge, and mastery worsened with the increase in extraversion; with the continuous increase in extraversion, the mastery level would improve contrarily. They made some conjectures about the reasons for this U-shaped relationship: on the one hand, based on the Eysenckian arousal theory [75], they believed that students with low levels of extraversion usually tended to seek a quiet learning environment (e.g., the library). This environment would be helpful to improving medical knowledge. On the other hand, according to Widiger and Mullins-Sweatt [76], high extraversion might be associated with a serious, methodical learning approach, and this learning approach would also help to master knowledge. The researchers showed that medical students with moderately high levels of neuroticism (or low emotional stability) performed better in clinical skills [12]. They proposed a possible explanation for the positive correlation between neuroticism and clinical skill. Based on previous research, they believed that vigilance was a characteristic of moderately high levels of neuroticism (or low emotional stability) [76], and vigilance was one of the important factors affecting clinical success [77]. In other words, vigilance mediates the positive correlation between moderate neuroticism and clinical skills. As the scores on emotional stability, which were used to reflect neuroticism in this experiment, were not very low (low value in the normal range), this positive correlation was only effective within a certain range. 

Ferguson et al. also found that conscientiousness enhances medical knowledge acquisition but reduces the acquisition of clinical skills [12]. Another experiment conducted with a group of medical students at Nottingham Medical School reached the same conclusion [78]. This experiment, which recorded and analyzed the relationships between performance and personality traits, showed that highly conscientious students were more likely to achieve good results in medical knowledge assessments but have poor clinical skills assessment outcomes (e.g., OSCE). The researchers speculated that the relationships between performance and personality traits change with the environment. In the pre-clinical years, medical students are taught in the classroom or laboratory. In this stable and calm environment, behaviors related to high conscientiousness (e.g., systematicness and organization) would help students form systematic learning methods to gain medical knowledge. However, students needed more flexibility [79] and adaptability for facing the high-pressure environment brought about by clinical years [12]. In this context, the rigidity of thinking associated with a high level of conscientiousness may not be conducive to learning clinical skills. In other words, conscientiousness was positively related to mastery of medical knowledge but negatively related to clinical skills. However, another experiment involving 703 UCL medical students concluded that higher conscientiousness could predict higher OSCE (objective structured clinical examination) scores [80]. 

As the influences of the same personality traits on the mastery of medical knowledge and clinical skills may change with changes in the learning environment (from pre-clinical to clinical years), and in order to predict the performances of medical students and have timely interventions for poor performance, medical educators should thoroughly understand the personality traits of students and adjust the intervention measures according to the specific situations.

### 3.6. Academic Performance

Numerous studies have examined how personality traits predict academic performance. Meta-analyses of educational research based on a five-factor model have also shown an association between personality and academic performance. Conscientiousness, openness, agreeableness, and extraversion positively correlate with academic performance [81,82]. Generally, individuals with higher conscientiousness are considered success-oriented, planned, organized, trustworthy, and responsible [83]. Openness is a personality trait related to creativeness, intellectual curiosity, and open-mindedness. Agreeableness can be expressed as an individual’s level of collaboration, temperateness, trustworthiness, and flexibility. Individuals with higher agreeableness prioritize success in the work/school environment and social activity and tend to attempt solving the problems they encounter [84]. Individuals with extraversion traits, especially when enthusiastic, cheerful, assertive, energetic, enterprising, and excited about prospects, tend to efficiently develop social interactions for improving the motivation and commitment of their colleagues [85]. One study provides evidence for the role of personality in cooperative group work in flipped classrooms (FC). The influences of personality traits varied according to gender, motivation, interaction, and engagement. The most striking conclusion of this study is that extraversion is the personality trait with the greatest positive effect on academic success in collaborative FC. This finding demonstrates that students with high extraversion might tend to utilize FC to improve their academic performance [86]. In addition, the research carried out by Komarraju et al. among 308 college students showed that students who got higher scores in agreeableness and conscientiousness could get higher GPAs, and the level of neuroticism was negatively related to GPA [87]. Therefore, it is useful to study personality factors in medical students and assess factors influencing academic performance [88]. 

Some studies have provided evidence supporting the role of personality traits in predicting the success of medical students’ academic performance [89,90]. Conscientiousness, neuroticism, and agreeableness were considered the predictive success criteria across the study years of dental school [90]. Openness was significantly related to the aspects of clinical education; however, this relationship was negative. A facet of openness and ideas, together with positive emotions and a facet of extroversion, improved the prediction of performance in clinical studies. Those who were less open to new ideas, that is, those who tended to focus narrowly on a limited number of topics, performed better in year 2 and 3 coursework and year 3 clinical work. However, positive emotions predicted third-year clinical training, and students with more positive emotions performed better in the clinical components of dental training [91]. Conscientiousness was considered the best predictor of academic education and clinical training success [92,93]. The facets of conscientiousness, competence, dutifulness, and achievement were strongly positively related to the success criteria, and order and self-discipline predicted better academic achievement but did not predict clinical performance [92]. Currently, most medical studies have been limited to investigating the effects of personality traits on academic performance. Further investigation is needed to explore how personality traits of medical students interact with study behavior or course programs to achieve success in academic performance and clinical training.

### 3.7. Career Planning 

The choice of specialty in medicine is an important decision for the health system, and for medical students. Career planning of medical students needs to combine the individual reasons, professional desires, and needs of the health system. These individual factors include gender, economic status, personality, personal interest, mentoring from a professor, clinical experience, expected income, family influence, lifestyle, and the influence of public media [13]. A cross-sectional study reported that gynecology and surgery were significantly positively associated with male medical students. This capacity to contact patients has also trended toward gynecology and surgery. However, the absence of a life-threatening emergency and advantageous exercise hours was positively associated with their choice of fundamental science. The choice of fundamental science specialties is associated with envy toward access to a university career [94]. In medical schools, where relatively high percentages of graduating seniors were planning their academic careers, students reporting mistreatment experiences were less likely to be planning careers in academic medicine (marking either basic science teaching/research or clinical discipline teaching, research, and patient care) [95]. 

Personality traits, as important intrinsic factors, can also participate in the specialty choosing of medical students. Lydon et al. examined whether personality differed based on gender, level of training, or medical specialty among 200 physicians and 134 medical students. Post-internship doctors scored significantly higher on conscientiousness than those pursuing basic medical training. Among those pursuing basic medical training, women scored significantly higher than men on agreeableness and conscientiousness. Among the post-internship respondents, females scored significantly higher on agreeableness. Among those pursuing basic medical training, those interested in person-focused medical specialties (those with an inclination toward people and the entire patient, including general practice, internal medicine, obstetrics and gynecology, pediatrics, and psychiatry) scored significantly higher on extraversion and conscientiousness and lower on neuroticism than those who had no strong preference [96]. A questionnaire survey of year 4 medical students (*n* = 110) in July 2015 illustrated that more agreeable students preferred clinical medicine to basic medicine, and more open students preferred medical departments to others (e.g., surgical, emergency medicine, radiology, and laboratory medicine). Personal interest was a significant motivational factor in more agreeable and conscientious students [13]. The BMJ Group performed a cross-sectional study at King Khalid University Medical School, including 590 students during the 2010–2011 academic year. A long version of the Zuckerman–Kuhlman personality questionnaire, which measures five personality factors, was used. It reported that male students had significantly higher scores on the “impulsive sensation seeking” scale, and students preferring a surgery specialty had the highest scores on the “impulsive sensation seeking”, “neuroticism–anxiety”, “aggression–hostility”, and “sociability” scales [97]. Despite these limitations, these studies might be helpful to medical students, tutors, and educators in the specialty choice process. Further research with a larger number of students will be required to evaluate the relationships between personality traits and the specialty choices of medical students.

## 4. Conclusions

Personality traits can affect medical students’ lives and work. Among them, personality traits generally regarded as positive, such as conscientiousness, extroversion, openness, and agreeableness, play positive roles in the life and work of medical students. Students with these traits usually have better health outcomes and life performance, higher life satisfaction, better doctor–patient relationships with patients, and better academic performance than those with high levels of neuroticism. However, neuroticism is the only personality trait that positively impacts perceptions of disease risk. In terms of career planning, students with high levels of agreeableness were more likely to take clinical medicine; more open students preferred surgical, emergency medicine, radiology, and laboratory medicine; and medical students who scored higher in extraversion and conscientiousness tended to choose general practice, internal medicine, obstetrics and gynecology, pediatrics, and psychiatry. Conscientiousness enhanced medical knowledge acquisition but reduced the acquisition of clinical skills, and there was a U-shaped relationship between extroversion and mastery of medical knowledge. Conversely, medical students with moderately high levels of neuroticism performed better in terms of clinical skills. 

Therefore, in the future, given the complex relationships between personality traits and the life and work of medical students, it is suggested that researchers in this field should further explore and make it clear whether they can be analyzed through quantitative data. This means that after a certain personality trait gets a certain score in a test, it should be directly judged whether it has a positive or negative impact on the medical student. In particular, the researchers are supposed to pay attention to the changes in the same personality traits in different situations. The theoretical results about the influences of personality traits on medical students should be fully applied to the practice of medical education, and the theory should be further improved according to the exploration results. Then, the perfect theory should be used to guide medical education and related training, which should focus on improving the beneficial personality traits of medical students. 

On the other hand, because the frequently used measurement methods of personality trait measurement rely on self-report questionnaires, the final results may be biased, because the options given by these volunteers participating in the experiments may not reflect their actual situations in all cases. Therefore, some researchers believe that the Big Five tests do not create accurate personality profiles. In addition, some psychologists also disagree with this model because they believe it ignores other areas of personality. The above situation may lead to completely different results for experiments on the same content. This review lacked a discussion on this possible issue, and on the impacts of personality characteristics on medical students’ religious beliefs and political identities. Making up for the above limitations will be one of the focuses of our next work.

In the future, our research will focus on how to apply the conclusions of this review to practical medical teaching and design relevant teaching courses based on the personality characteristics of medical students. Finally, medical students can improve their personality traits and achieve better performance in life and work.

## Data Availability

The data that support the findings of this study are available from the corresponding author upon reasonable request.

## Supplementary Materials

The following supporting information can be downloaded at: https://www.mdpi.com/article/10.3390/ijerph191912376/s1, Table S1: The methodological characteristics of the research-type studies included in this review.
